# An Instance Segmentation Model for Strawberry Diseases Based on Mask R-CNN

**DOI:** 10.3390/s21196565

**Published:** 2021-09-30

**Authors:** Usman Afzaal, Bhuwan Bhattarai, Yagya Raj Pandeya, Joonwhoan Lee

**Affiliations:** Division of Computer Science and Engineering, Jeonbuk National University, Jeonju 54896, Korea; usman.afzaal45@gmail.com (U.A.); bhubon240@gmail.com (B.B.); yagyapandeya@gmail.com (Y.R.P.)

**Keywords:** instance segmentation, smart farming, convolutional neural network, strawberry disease detection, Mask R-CNN

## Abstract

Plant diseases must be identified at the earliest stage for pursuing appropriate treatment procedures and reducing economic and quality losses. There is an indispensable need for low-cost and highly accurate approaches for diagnosing plant diseases. Deep neural networks have achieved state-of-the-art performance in numerous aspects of human life including the agriculture sector. The current state of the literature indicates that there are a limited number of datasets available for autonomous strawberry disease and pest detection that allow fine-grained instance segmentation. To this end, we introduce a novel dataset comprised of 2500 images of seven kinds of strawberry diseases, which allows developing deep learning-based autonomous detection systems to segment strawberry diseases under complex background conditions. As a baseline for future works, we propose a model based on the Mask R-CNN architecture that effectively performs instance segmentation for these seven diseases. We use a ResNet backbone along with following a systematic approach to data augmentation that allows for segmentation of the target diseases under complex environmental conditions, achieving a final mean average precision of 82.43%.

## 1. Introduction

Crops are vulnerable to a variety of diseases leading to major production losses in the agriculture sector around the globe [1]. To increase crop quality, it is of prime importance for plants to be protected against any potential diseases. This also has the effect of reducing the cost of food production. To achieve these goals, the options available include traditional methods of identifying and diagnosing plant diseases. These include inspections carried out by a professional farmer or the examination of an affected sample in the laboratory. It is however clear that such a manual inspection-based approach is not only time consuming and expensive but also requires a high level of domain knowledge by an expert. Furthermore, not all such methods are particularly accurate and therefore may fail to successfully diagnose the plant disease at hand [2]. Another solution is the use of pesticides during food production, but the use of potentially harmful chemicals leads to lower food quality. Moreover, it also increases the labor cost. In summary, there is a need for plant disease diagnosis that is effective yet practical considering the deployment costs. To that end, an automatic disease detection system that can not only identify the type of plant disease but is also able to identify the exact location of the lesion would be most suitable.

With recent advances in deep learning, many reliable automatic systems have surfaced that excel at accurately diagnosing various types of crop diseases [3]. These systems can help reduce the time and effort required in crop disease identification when compared with a manual diagnosis wherein an individual with certain expertise is required to inspect a suspected area. There also exist multiple visual challenges in properly analyzing the suspected area, for example, varying illumination conditions, multiple object sizes and variations in background, etc. Moreover, even if a successful identification is made, there is another task of properly classifying that disease. Thus, vision-based deep learning systems will be best suited to this problem. At present, two types of protocols are followed when using these systems: (1) A person is required to go around a greenhouse and take pictures of the suspected areas manually using a handheld camera device. Next, the images taken are processed using automatic detection systems. (2) Robots perform surveillance in the entirety of the greenhouse and collect required photos autonomously, which are subsequently processed by an automatic disease detection system [4].

With this, we understand that deep learning-based systems are a superior choice over traditional methods for crop disease diagnosis as they are economical, feasible and accurate under variable conditions. In this paper, we specifically focus on autonomous disease detection for strawberries. We propose a deep learning algorithm for locating, classifying and determining the precise location of the diseases in strawberries. It is understood that deep learning requires a large amount of data for properly training the algorithm such that the problem of overfitting is avoided. Since gathering an adequately large amount of data can be difficult, various data augmentation techniques are available for developing powerful models even with limited data [5]. Our model is based on the widely used Mask R-CNN approach [6] which allows efficient fine-grained instance segmentation of multiple strawberry diseases. We report results for two different backbones for our experiments, the ResNet50 and ResNet101 [7]. Without using any augmentation techniques, we achieve a mAP of 79.84% and 80.24% for ResNet50 and ResNet101, respectively. We then instrument a number of augmentation techniques and select the highest performing augmentation methods for our dataset. With an improved learning strategy, we achieve a final mAP of 82.43% on the ResNet101 architecture.

### Contribution

Our main contributions are three-fold.

1. We introduce a new dataset towards advancing the current state of research in instance segmentation systems for predicting strawberry diseases.
2. We then propose an optimized model based on the Mask R-CNN architecture to effectively perform instance segmentation for seven different categories of strawberry diseases.
3. We investigate a range of augmentation techniques to determine the most suitable augmentations for our novel dataset.

The rest of this paper is organized as follows: In Section 2, a thorough review of related methods available in the literature applied to the plant disease detection problem is arranged. In Section 3, expansive information on our dataset and the network architecture utilized is provided. Next, in Section 4, the experimentation methodology employed and the results obtained thereof along with the conclusion drawn are presented. The paper concludes with a brief review of the conducted study in Section 5.

## 2. Related Work

In this section, we provide a basic overview of the classical and modern deep learning-based techniques for image analysis in the context of plant disease recognition. Note that a comparison with related work is also reported at the end of Section 4.6 after the presentation of our results.

### 2.1. Classical vs. Deep Learning-Based Approaches

Classical computer vision techniques are well established and optimized. These involve feature exploration of an image where an expert-designed feature descriptor is used. Various hand-crafted feature descriptors that are able to provide semantic and robust representations of the input images along with a number of visual feature classifiers have been developed for a number of problem domains [8]. Regardless, extracting very deep and complex features is difficult using these traditional approaches [9]. In the context of plant disease detection, several characteristics of the plant diseases are used to design the imaging scheme and to capture images with uniform illumination. This reduces complications at the expense of increased application cost.

Nonetheless, it is still not possible for conventional algorithms to satisfactorily eliminate the effects of scene variations including, but not limited to, noise, background clutter and scale variations, all of which adversely affect system performance [3]. Typical examples of visual feature extractors include the Scale Invariant Feature Transform (SIFT) [10], the Histogram of Oriented Gradients (HOG) [11] and the Haar [12]. Machine learning algorithms are used on top of such features to make the representations more hierarchical and informative and to develop classifiers for visual detection. Examples of such algorithms include Support Vector Machine (SVM) [13], AdaBoost [14] and Deformable Part-based Model (DPM) [15].

On the contrary, deep learning-based approaches achieve much higher performance compared to traditional computer vision in the problems of classification and detection. Deep learning brings forward the concept of end-to-end learning in which an algorithm is fed a large number of images that are annotated with the object classes [16]. The need to design customized features is eliminated as the neural network automatically discovers the underlying patterns in the classes present and works out the most expressive and important features for each category. These features are extracted using the multiple hidden layers in a deep neural network where high-level features can be obtained by the combination of low-level features extracted by each layer. The classifier is learned jointly while training the same network using these extracted representations which now contain the global and contextual features of the input images [3]. However, the superior performance of deep learning models comes at the cost of increased training time in addition to increased computing and data requirements. We opted for a deep learning-based approach for our experiments.

### 2.2. The Problem of Detection

It is vital to correctly identify and localize symptoms of the disease. There are a number of vision-based approaches that can be used to detect plant diseases. These approaches can be based on image classification, object detection or image segmentation.

#### 2.2.1. Classification Approaches

Classification means determining the category information of an input image without specifying the location of the object detected. An algorithm trained to recognize certain plant diseases is fed images of affected plants after manual inspection for possible symptoms. The model then predicts the category of the disease present. Although the symptoms can be classified without the need of an expert, the localization of the diseased part still requires human intervention. As regards the imaging scheme, object-centric images were focused in earlier applications for classification [17].

Table 1 summarizes deep learning-based classification approaches that have been used to solve similar problems. A Convolutional Neural Network (CNN), such as AlexNet [18], ResNet [7], ResNeXt [19], Inception V4 [20], EfficientNet [21] and HRNet [22], is commonly used as the feature extractor for such purposes. For most applications, the deep neural network is initially trained on the ImageNet dataset of the ImageNet Large-Scale Visual Recognition Challenge (ILSVRC) [23], and these pre-trained weights are then later fine-tuned for the given application since training a neural network from scratch is often not feasible. Fang et al. [24] designed a network based upon ResNet50 for plant diseases and pest detection. Traditional classifiers can also be used on top of features obtained via CNNs for classification [25,26]. Yalcin et al. [27] used SVM classifiers with different kernels and feature descriptors like LBP and GIST on features extracted using a CNN. Other than the prediction of image level labels, a classification network can also be used to obtain location of the disease via pixel-by-pixel classification. For the problem of maize disease detection, Dechant et al. [28] trained a CNN to generate a heatmap that shows the probabilities of infection for each region. These heatmaps were finally used to classify complete images into regions containing and not containing infection leaves.

#### 2.2.2. Detection Approaches

Object detection approach aims at predicting the class information of the objects present in an image together with the exact locations of the symptoms. This is accomplished by drawing a bounding box around the affected area which eliminates the need for human intervention. Contrary to image classification, object detection provides more flexibility with regards to the direction and the distance the input images are captured. The basic framework of object detection models can be divided into two main types: one-stage and two-stage methods. Two-stage models consist of a Region Proposal Network (RPN) that generates a set of candidate regions which are classified into different object categories by the later stage of the network. These regions used by the RPN are extracted using feature maps generated by a CNN. One-stage networks treat object detection as a regression or classification problem and output bounding boxes and classification results of the unified network. The two-stage approach has been constantly improved to reduce the detection time with the aim of increasing the practicality of the system, but the inference speed is still slower compared to the single-stage detection systems as these networks are computationally less expensive than their two-stage counterparts. Notable examples for the two-stage approach include R-CNN [33], Spatial Pyramid Pooling (SPP) [34], Fast R-CNN [35], Faster R-CNN [36], Feature Pyramid Network (FPN) [37] and DetectoRS [38]. On the other hand, the one-stage approach includes EfficientDet [39], YOLO [40], CenterNet [41] and the recent Transformer-based approach, Swin-Transformer [42].

Table 2 provides a detailed summary of plant disease recognition based upon object detection models. Contrary to image classification, data annotation is more expensive for detection because bounding box locations must be provided for each object instance for all the images contained in the dataset. Due to the lack of large datasets for object detection pre-training, the network backbone in detection is also pre-trained on the ImageNet Dataset or in some cases, the Microsoft Common Objects in Context (MS-COCO) dataset [43]. The multi-stage approach has been used in various detection models for plant diseases [1,44,45]. Ozguven et al. [46], for instance, proposed a model based on Faster R-CNN for the detection of beet leaf spot disease. Moreover, Nie et al. [47] used attention mechanism with Faster R-CNN for verticillium wilt detection in strawberries.

#### 2.2.3. Segmentation Approaches

Segmentation is a high-level task which is considered one of the key problems in the field of computer vision. Segmentation leads to a complete understanding of the scene. The predictions produced by a segmentation model are much more fine-grained in comparison to a classification or a detection network. This is because the goal of segmentation is to label each pixel in an image with the corresponding class. A segmentation network can convert the task of detection into semantic and instance segmentation. In semantic segmentation, we do not differentiate between multiple instances of the same category, whereas in instance segmentation, the model will make distinctions between different objects belonging to the same category in case they are present together in a single image. Segmentation networks can be broadly divided into Fully Convolutional Networks (FCN) [49] and Mask R-CNN. FCN-based networks initially extract the features of the input images using CNNs and then gradually restore the feature size to that of the input image using upsampling or deconvolution layers. Traditional FCN and SegNet [50] are typical examples. On the other hand, Mask R-CNN is a popular instance segmentation model in which multiple objects of the same category can be differentiated and counted, even in the case of overlap. More examples include TensorMask [51] and YOLACT [52].

Table 3 summarizes the different model designs for the segmentation of lesions and normal areas in plants. In comparison to object detection, the process of data annotation for segmentation is quite expensive because it requires providing the algorithm the exact shape of the objects present, in addition to their location. Because of the inherent difficulty of gathering pixel-level labeled datasets for segmentation, their scale is not comparable to that of the classification datasets [53]. For this reason again, many algorithms are pre-trained on the classification datasets or the MS-COCO dataset before being fine-tuned for the required task. In practice, Stewart et al. [54] used Mask R-CNN to detect maize northern leaf blight (NLB) disease using autonomous aerial vehicle images. Wang et al. [55] developed a system based on Mask R-CNN to segment diseases in tomatoes. Similarly, in the field of agriculture, Khan et al. [56] proposed a cascaded encoder–decoder (CED-Net) architecture for detecting precise locations of weeds and crops on farmland [57].

From the three available vision-based recognition methods, we have selected the instance segmentation approach for our problem because of its ability to provide more fine-grained predictions compared to the other two methods. This is in addition to its ability to differentiate between multiple instances of the same class. The proposed strawberry disease detection system is based on the two-stage Mask R-CNN architecture.

## 3. Materials and Methods

**Figure 1:**
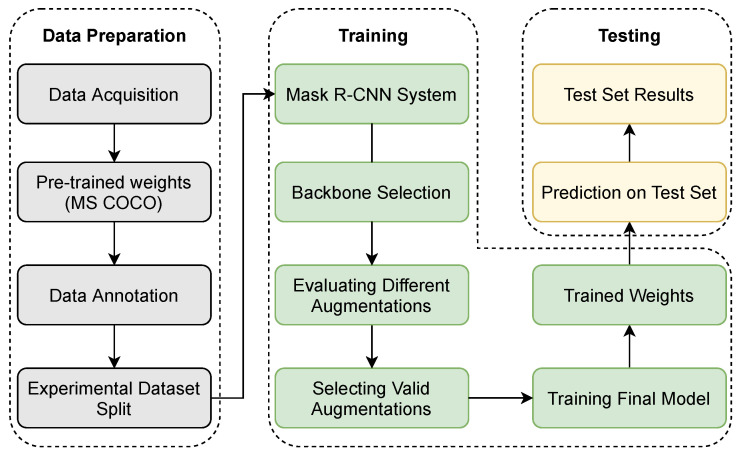
Flowchart for developing a system to detect strawberry diseases.

We used Matterport’s public Tensorflow implementation of the feature pyramid network-based Mask R-CNN for our experiments with appropriate hyperparameter modifications [60]. The design flow of our approach is shown in Figure 1. The first step is to arrange a suitable dataset. We annotated our dataset with Labelme, an open-source image annotation tool. The dataset was augmented with different kinds of augmentation techniques using the python library Imgaug which allows data augmentation by altering properties of the images such as geometry, color, arithmetics, etc. An optimized augmentation graph was used to train the Mask R-CNN model to obtain the final mask predictions. Detailed descriptions of each module will be provided in their respective sections below.

### 3.1. Dataset

The literature indicates a scarcity of datasets pertaining to the instance segmentation of different kinds of strawberry diseases. Although various models have been developed to perform object detection for multiple diseases in strawberries [4,47], there is much to be desired when it comes to datasets allowing fine-grained instance segmentation of multiple diseases and pests in strawberries. In an attempt to fill that void, we introduce a new dataset that allows users to segment seven different kinds of strawberry diseases. Since our dataset consists of images that are collected in real fields/green houses instead of a laboratory, it introduces multiple challenges such as having background variations, complex field conditions, different illumination settings, etc. As a result, these variations allows us to design models that have a higher capacity to be more robust and generalizable.

**Figure 2:**
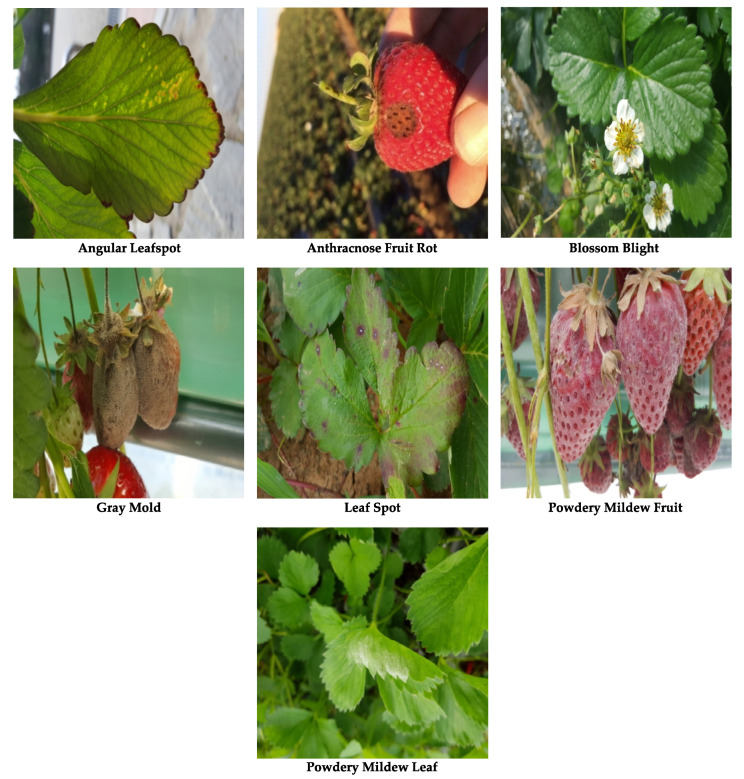
The seven types of strawberry diseases that our model is trained to detect.

The dataset contains 2500 images for strawberry diseases collected from various greenhouses using camera-equipped mobile phones. The data was collected from multiple greenhouses under natural illumination conditions in South Korea to ensure a diversity of environmental factors. The diseases were verified by experts in the field. Note that approximately 20% of the images contained in the dataset were collected from online sources (Università di Bologna, Bugwood.org (accessed on 22 July 2021); Ontario Ministry of Agriculture, Food and Rural Affairs (OMAFRA); Nicole Ward Gauthier, University of Kentucky; Gerald Holmes, Strawberry Center, Cal Poly San Luis Obispo, Bugwood.org; William W. Turechek USDA ARS; Frank J. Louws, NC State University; Steven Koike, Plant Pathology Farm Advisor, University of California Agriculture and Natural resources blogs; Garrett Ridge, NC State University; Cornell University; College of Agriculture and Life Science blogs; Madeline Dowling, phytographics.com (accessed on 22 July 2021); Jonas Janner Hamann, Universidade Federal de Santa Maria (UFSM), Bugwood.org; Clemson University—USDA Cooperative Extension Slide Series, Bugwood.org; University of Georgia Plant Pathology, University of Georgia, Bugwood.org; Paul Bachi, University of Kentucky Research and Education Center, Bugwood.org; Scott Bauer, USDA Agricultural Research Service, Bugwood.org; John Hartman, University of Kentucky, Bugwood.org; more details in dataset.txt.) [61,62,63,64,65,66]. The images in the dataset are processed to be of resolution 419 × 419. With regards to imaging distance, the dataset provides both close-up and distant views of the diseases. The dataset is composed of seven different types of strawberry diseases, with images ranging from initial, middle and final stages of the diseases. An example case for all seven strawberry diseases is visualized in Figure 2. The dataset is split into 1450, 307 and 743 images for training, validation and test sets, respectively. Table 4 provides a brief summary of our dataset. Online augmentation methods are used and as a result, the final number of images depends on the number of epochs the model is trained on the dataset. The image augmentation used here is described in more detail in Section 4. The dataset will be made publicly available for further experimentation.

### 3.2. Mask R-CNN Architecture

As mentioned above, we based our detector on the widely used Mask R-CNN model. Mask R-CNN is a natural update to the previous Faster R-CNN system. It is a simple yet efficient algorithm. It enables instance segmentation for a multitude of applications.

**Figure 3:**
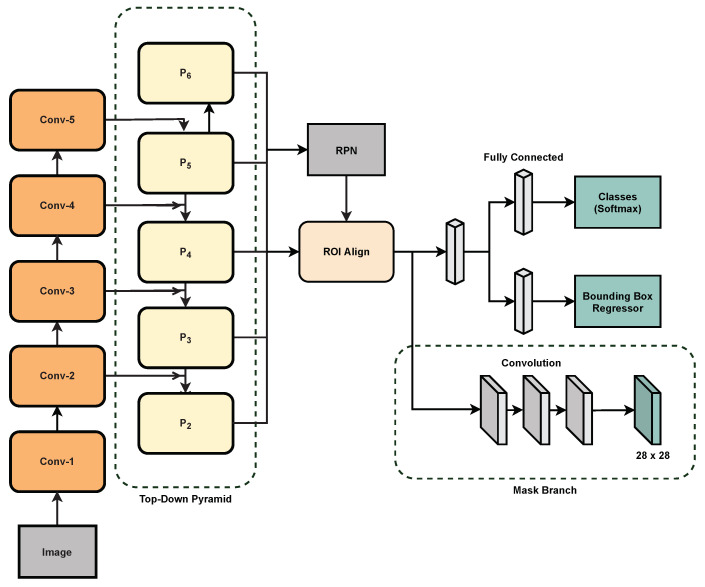
ResNet and Feature Pyramid Network (FPN)-based disease detection model.

Mask R-CNN brings together Faster R-CNN and FCN for both object detection and instance segmentation. The overall architecture is presented in Figure 3. For our final model, we first use a ResNet101 MS-COCO pre-trained backbone for extracting the feature maps from an input image. Treating the extracted features as the bottom-up pyramid, the top-down feature pyramid is generated using lateral connections to obtain multi-scale, high-level semantic feature maps. The extracted feature maps are then used by a Region Proposal Network for generating Regions of Interest (ROIs) on an image. In the RPN, a small network slides on the output feature map of the backbone, and each sliding window is mapped to a lower-dimensional feature vector. This feature vector is the input to two parallel fully-connected layers, one of which is responsible for outputting the locations of the region proposals while the other one judges if there is a target object in the region box or not. For k number of region proposals, these regression and classification layers are realized though a 1 × 1 convolution filter resulting in 4 k and 2 k output values for the regression and classification layer, respectively. These k region proposals are parameterized relative to reference boxes known as anchors.

In Mask R-CNN, an anchor is centered at the sliding window and has five different scales, one for each of the five levels in the feature pyramid. It also has multiple aspect ratios for every scale. We used the default values for the scales and the aspect ratios as recommended in the original paper [6]. The anchor scales are {32^2^, 64^2^, 128^2^, 256^2^, 512^2^} pixels on the {P2, P3, P4, P5, P6} feature maps in the top down pyramid, respectively, whereas the aspect ratios for the anchors are {1:2, 1:1, 2:1}. The RPN generates a huge amount of region proposals which may overlap for the same objects. Therefore, to reduce the number of generated region proposals, Non-Maximum Suppression [67] was utilized. In the end, the remaining region proposals were sorted according to their classification scores and a subset was selected for further processing.

According to the size and position of the region proposals, these ROIs are then assigned to different scales in the pyramid of the features. In essence, these ROIs are clipped from the feature maps and are passed into an ROI Align layer. Using ROI Align, a small feature vector of a fixed size (7 × 7 in this work) was extracted from each ROI and sent into the heads of the network. The first head predicts the classification result of the boxes while the second one provides the regression output which gives the coordinates of the region proposals. These results were realized by passing the fixed-length vector into two parallel fully-connected layers. The third and the final branch of the network predicts segmentation masks of the detected objects. These masks were acquired by an FCN-based architecture using an ROI pool size of 14 × 14 instead of 7 × 7. It is later upsampled to a size of 28 × 28 for generating the final predicted masks.

### 3.3. Evaluation Metrics

The proposed model is evaluated using the metric of mean average precision (mAP) introduced by the PASCAL VOC Challenge [68]. For mAP, the precision and recall are computed, leading to a precision-recall curve. The Average Precision (AP) is the area under the precision and recall curve for detection. The equation for precision and AP is as follows:

(1) $$Precision=\frac{True\;Positives}{True\;Positives+False\;Positives}$$

(2) $$AP=\frac{1}{11}\sum_{r\epsilon \left\{0.0,\cdots ,1.0\right\}}^{}P_{interp}\left(r\right)$$

where,

(3) $$P_{interp}\left(r\right)=\max_{\tilde{r}\geq r}p\left(\tilde{r}\right)$$

Here, $P_{interp}\left(r\right)$ represents the maximum precision value for any recall value greater than *r*, whereas *P*($\tilde{r}$) is the actual precision at recall $\tilde{r}$. True Positives (TP), False Positives (FP), True Negatives (TN) and False Negatives (FN) are determined using a parameter called Intersection over Union (IoU). IoU is based upon the overlap of a predicted mask with the ground truth mask. Following PASCAL VOC, a prediction in our case is positive if IoU ≥ 0.5. The equation for IoU is:

(4) $$IoU=\frac{Area\;of\;Intersection}{Area\;of\;Union}$$

We first computed the average precision for each image and then computed the mean of all the values to obtain the final mAP.

### 3.4. Multi-Task Loss

A multi-task loss function is defined on each sampled RoI during training as:

(5) $$L=L_{class}+L_{box}+L_{mask}$$

The class and the box loss can be calculated as follows:

(6) $$L\left(p,u,t^{u},v\right)=L_{class}\left(p,u\right)+\lambda \left[u\geq 1\right]L_{box}\left(t^{u},v\right)$$

where $L_{class}\left(p,u\right)=-logp_{u}$ is the log loss for the true class *u* and $p=(p_{0},\cdots ,p_{k})$ is the discrete probability distribution (per ROI) over K + 1 categories, which is computed using softmax. The smooth L1 loss is used for *L_box_*, which can be ignored by the indicator function, is defined as:

(7) $$\lambda \left[u\geq 1\right]=\left\{\begin{array}{cc} 1 & if\;u\geq 1\\ 0 & otherwise \end{array}\right\}$$

The mask head has a Km^2^ dimensional output as it generates a mask of size *mxm* for every ROI and each of the K classes. For this, a per-pixel sigmoid and a binary cross-entropy loss is used. For an ROI associated with a ground-truth class, *L_mask_* is only defined on the mask of that particular class.

## 4. Experimental Results and Discussion

### 4.1. Implementation Details

The initial experiments were performed without augmenting the dataset. For comprehensive evaluation, we performed experiments on two backbones, the ResNet50 and ResNet101. Both of the backbones were initialized with pre-trained MS-COCO ResNet101 weights. Since ResNet50 has fewer layers than ResNet101, for ResNet50 we only took weights of the corresponding layers from the pre-trained ResNet101 weights. We chose Stochastic Gradient Descent (SGD) as the optimizer with the learning rate set to 0.0001, a momentum of 0.9 and a weight decay of 0.0001. Batch size was set to 2 and the training was conducted on an Nvidia Titan XP GPU.

For the settings related to image size, we selected a value of 512 and 960 as the minimum and maximum image dimensions, respectively. Here, the maximum dimension value ensures that the longer side of an image does not exceed it. We resized and padded an input image with zeros to obtain a square final image of the aforementioned size. The number of validation steps and iterations in each epoch were set at 200 and 725, respectively. For both the experiments, all of the network layers were fine-tuned. In Table 5, we present the results of our preliminary experiments.

It is clear from Table 5 that at the same hyperparameter values, ResNet50 outperforms ResNet101 despite having a lesser depth and a weaker feature representation capability. We can conclude that it is due to ResNet101 slightly overfitting the training dataset.

### 4.2. Augmentation Graph

We picked ResNet101 model from Table 5 as the baseline for performing further experiments in order to select the most optimal augmentation graph for our final model. We followed a systematic approach to determine what types and combinations of augmentations work best for the given dataset. The baseline model was trained on a number of different image augmentation techniques one by one, and the final results for each augmentation were noted. Each augmentation was applied on the training dataset with a probability of 0.5 in each iteration. We used the online image augmentation method. As a result, the final number of training images depends on the training period of the model. The final results are reported in Table 6. In the table, the Specifications column lists the hyperparameter choice as allowed by the augmentation library Imgaug. The *baseline* refers to the model with no augmentation. Some of the augmentations applied are a combination of the one applied previously. This time, they were applied simultaneously on each image. It can be observed that a number of augmentations improved the mAP of the model, whereas some also resulted in a decrease in the mAP. Such augmentations are considered unsuitable for our problem. A few augmentations led to minor improvements over the baseline.

### 4.3. Selection of Best Performers

**Figure 4:**
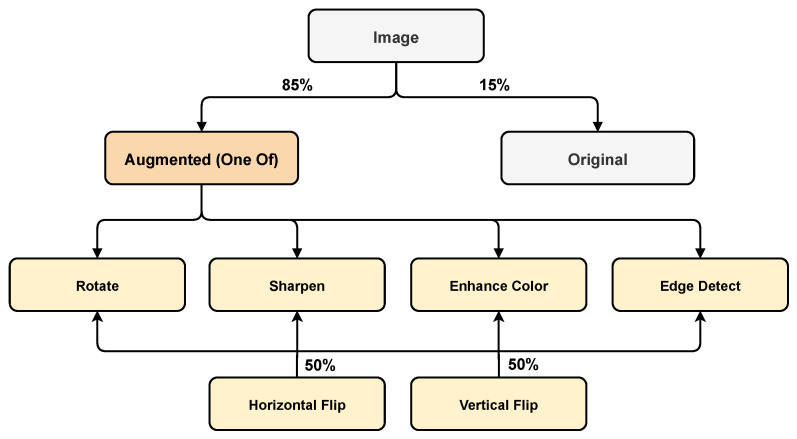
The selected augmentation graph.

**Figure 5:**
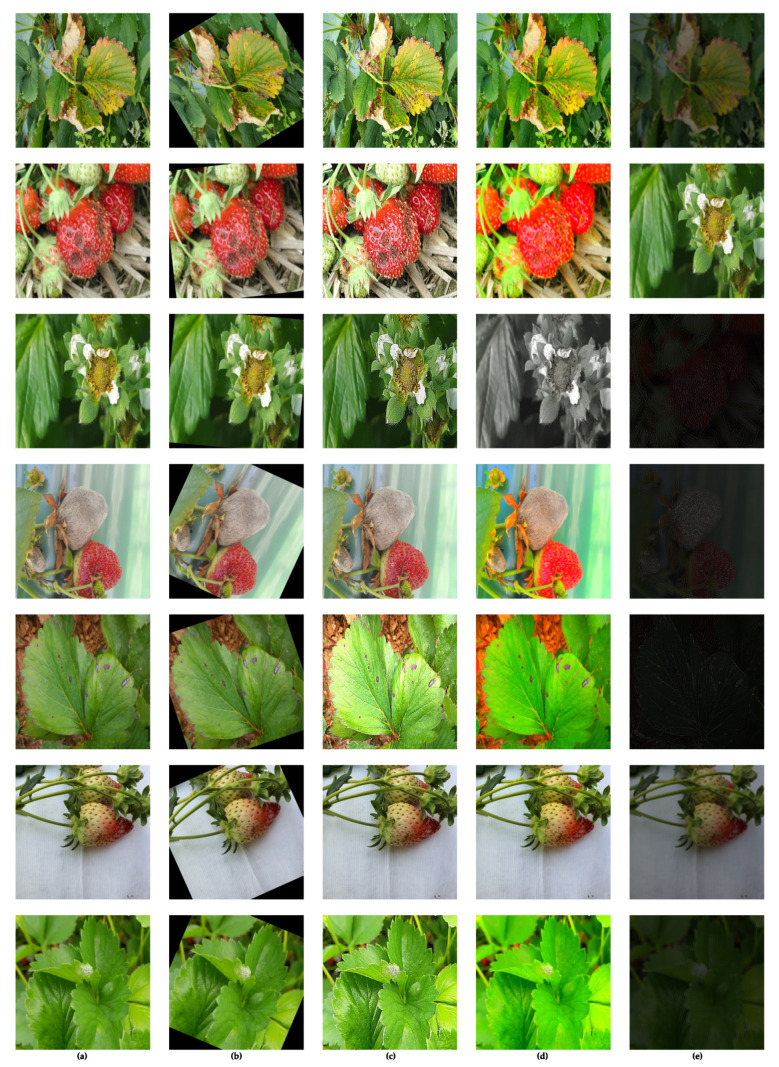
Visualization of the final augmentations selected for the dataset. (**a**) Original, (**b**) rotated, (**c**) sharpened, (**d**) enhanced color, (**e**) edge detect.

From Table 6, we observe that the high-performing augmentations when applied in a simultaneous fashion do not lead to substantial improvements over the baseline. Hence, for the final augmentation graph, most augmentations are picked from those that led to an improvement in mAP over the baseline. These are highlighted in bold text in Table 6. We applied the selected augmentations in each iteration individually with an application probability of 85% compared to 15% for no image augmentation. In addition, with each augmentation we used horizontal and vertical flipping with an application probability of 50%. Figure 4 illustrates the final augmentation approach. The best-performing augmentations for select images can be visualized in Figure 5 for each class.

### 4.4. Results on the Improved Dataset

We performed final experiments after applying the augmentation techniques selected above. The same protocols and hyperparameters as discussed in Section 4.1 were used except that an improved training strategy for further increasing the performance was also utilized. Training was performed using two GPUs with a batch size of 2 images per GPU, resulting in an effective batch size of 4. The model was trained for 50 epochs in total with an initial learning rate of 0.001 for 20 epochs, which was then decreased by a factor of 10 at the 21st and 41st epoch mark. During this period, every layer in the whole network was fine-tuned. After that, we further decrease the learning rate by a factor of 10, freeze the whole network and only fine-tune the heads of the network for five more epochs leading to a final mAP of 82.43% on our dataset.

Both ResNet101 and ResNet50 were trained using the updated training scheme. In the case of ResNet101 network, the augmentations alone are responsible for an increment of approximately 8.5% in the mAP. The final results are listed in Table 7 and Table 8.

### 4.5. Analysis of Model Predictions

**Figure 6:**
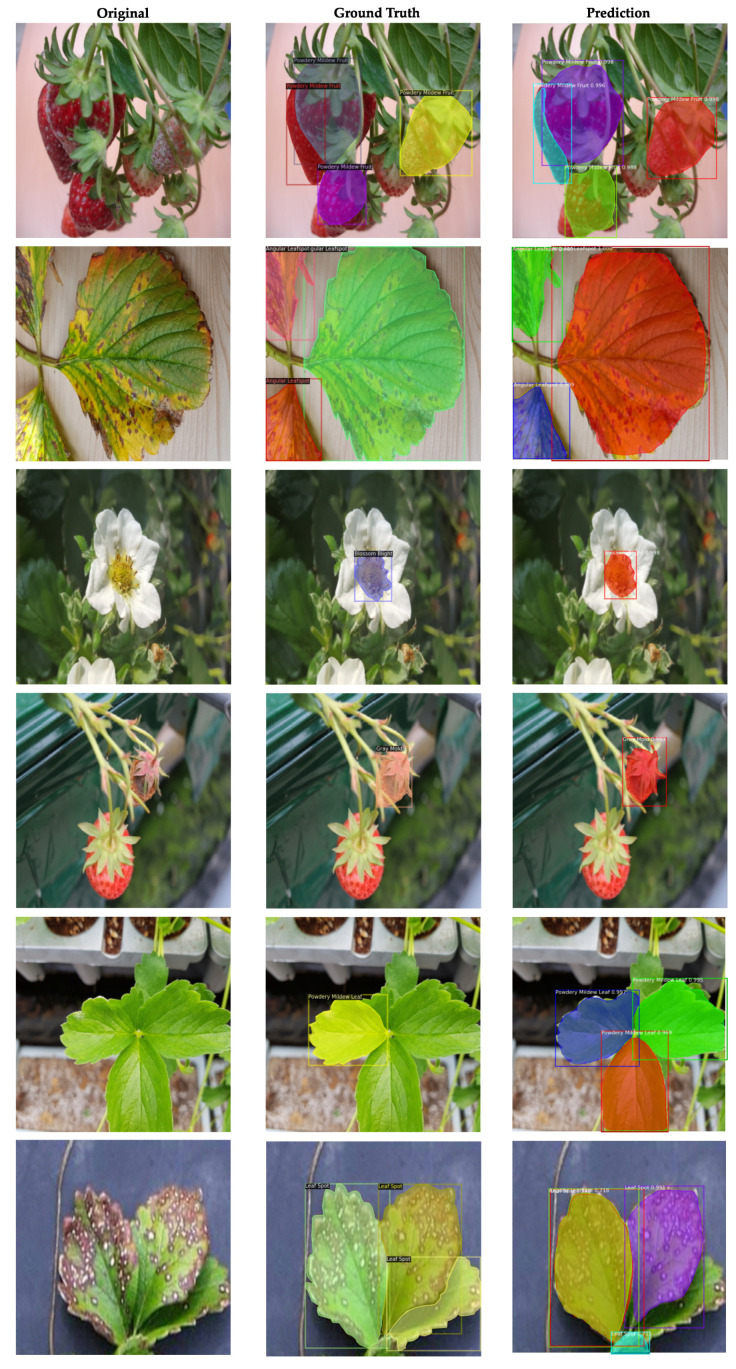
Predictions on the test dataset by the ResNet101 Network. The last two rows visualize some of the misclassifications done by our network.

**Figure 7:**
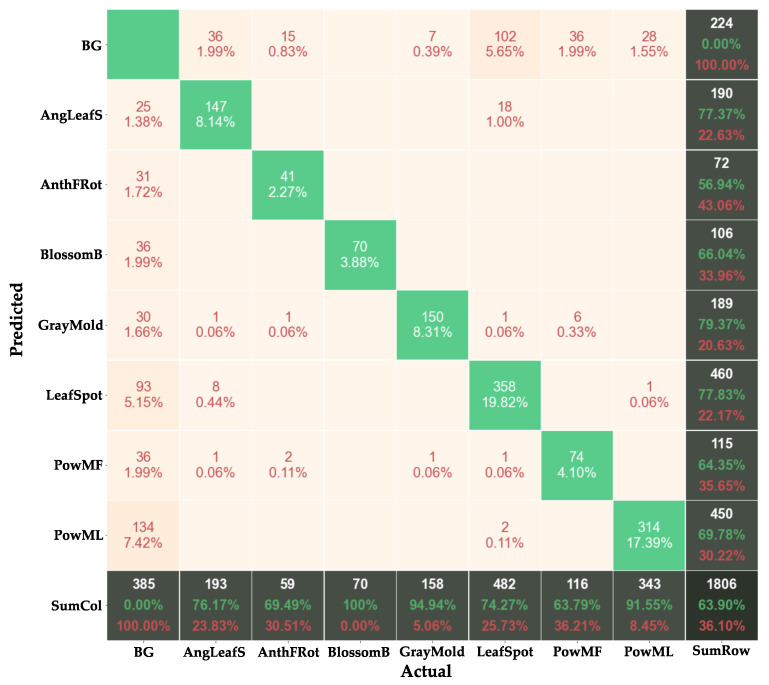
Confusion matrix of the Strawberry Diseases segmentation results (including Background, which covers the cases of model misses, i.e., detecting BG instead of an actual object or vice versa). Green Boxes: True Positives; Peach Boxes: Misclassifications (False Negatives, False Positives).

We visualize some of the final predicted masks by our model in Figure 6. Examples for both satisfactory predictions along with some misclassifications are presented. In Figure 7, we plot the confusion matrix for our final ResNet101 model to visually evaluate the performance of the detector. The matrix allows us to determine at what classes and features the neurons in the network mostly activate on. This enables us to identify inter-class confusions and to design rectifying procedures for the future. The x-axis in the matrix represents the ground truth class for each image, whereas the predictions done by the model on those images are shown on the y-axis. For instance, out of 158 instances of Gray Mold from the ground truth, 150 have been correctly detected by the model which is equivalent to 8.31% of the total predictions made by the model, while the model misclassifies 1 and 7 instances for Powdery Mildew Fruit and Background, respectively. To expand, the numbers on the edges denote the row and column-wise sum. For Gray Mold, the row and column-wise sum is 189 and 158, respectively. For the y-axis edge, 150 predicted True positives is equivalent to 79.37% of the total 189 Gray Mold predictions done by the network, leaving 20.63% as the error in this case. On the other hand, for the x-axis edge, out of total 158 Ground Truths objects for Gray Mold, 94.94%, or in other words, 150, have been correctly classified, leaving behind 5.06% or 8 as misclassifications. The bottom-right block is the sum of the row and column-wise totals, leading to a final value of 1806 objects. The sum of the True Positives is 1154, which equates to 63.90% of the total 1806 predictions.

Due to the complex patterns in each class, we observe that the system tends to be confused in various classes. Above all, the complicated background conditions seem to confuse the model, for the most part resulting in increased number of False Positives and False Negatives. Moreover, the network seems to slightly confuse the Leaf Spot class with Angular Leafspot, whereas a few Powdery Mildew Fruit instances are predicted as Gray Mold.

### 4.6. Disease Severity Level Analysis

**Figure 8:**
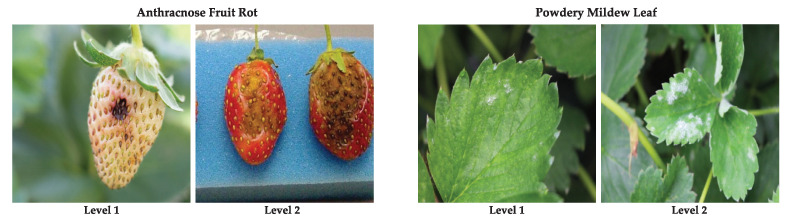
Examples for the diseases present in Level 1 and Level 2 for Anthracnose Fruit Rot and Powdery Mildew Leaf.

To evaluate the performance of the network for different degrees of infection, we divide our test dataset into two splits of 206 and 537 images with each split representing a specific level of disease severity. We name these splits: Level 1 and Level 2, where Level 1 denotes low-mid degree infection and Level 2 denotes a higher degree of infection. The splits are made after consulting with a domain expert on a number of features, including: (1) the severity of the disease present, (2) the spread of the disease, (3) and the maturity level of the leaf/fruit. In Figure 8, we visualize examples of both levels for two classes. Experiments are conducted on these dataset splits with the final Mask R-CNN ResNet101 network and the results, as reported in Table 9, show that the network is able to detect the diseases in each level with a high accuracy.

### 4.7. Comparison with Relevant Literature

Using the same dataset split as in the case of Mask R-CNN, we also report the results on YOLACT, a fully-convolutional real-time instance segmentation focused method in Table 10, with two different backbones, the ResNet101 and ResNet50. Every network is initialized with pre-trained imagenet weights. For training, we use a multi-GPU strategy where the maximum image dimension is set to 800 with an effective batch size of 8. SGD was used as the optimizer with an initial learning rate set to 0.001, a momentum of 0.9 and a weight decay of 0.0005. The model was trained for 55 k iterations and 40 k iterations for ResNet101 and ResNet50 backbones, respectively. The learning rate was decreased by a factor of 10 at the 25 k, 35 k and 45 k iteration mark, with the last one being applicable to the ResNet101 model only. A validation size of 200 images was used along with using random photometric distortions, image resizing and random flipping/mirroring/rotating as augmentations. COCO evaluation metrics are used to calculate the final mean average precision for the segmentation masks.

In Table 11, we compare results reported on other similar datasets. In their work, Ouyang et al. [69] performed basic segmentation for three kinds of strawberry diseases. The diseased strawberry fruit is first extracted using digital image processing and pattern recognition techniques, and then a comparison is made for the recognition and classification results of a neural network with SVM. No official accuracy is reported; rather, it is concluded that SVM has a higher recognition rate than the neural network when used as a classifier. Next, the dataset of Byoungjun et al. [4] report a basic mAP of 83.13% using Faster R-CNN with pre-trained ImageNet weights. Improvement is made using a cascaded architecture and pre-trained weights from PlantCLEF dataset. However, their dataset and thus the model is designed to perform coarse-grained object detection compared to fine-grained instance segmentation in this work. It is hoped that our model has the potential to match their final accuracy if a similar cascaded structure is employed, which we intend to explore as part of our future work.

## 5. Conclusions

Protecting plants from harmful diseases is key to maximizing yield and improving quality. Towards this goal, in this paper, we developed a deep learning-based model to autonomously detect and segment seven kinds of strawberry diseases. The dataset introduced in this work includes images taken under variable environmental conditions including variations in illumination settings, background, etc. Through empirical studies, we based our model on a superior feature extractor, suitable hyperparameter values, and best-performing augmentation techniques for the given dataset. We finally achieved a mAP of 82.43% on the test data. It is hoped that the dataset introduced herein along with the Mask R-CNN architecture-based instance segmentation model will contribute to solving the problems of plant disease detection. Future work will focus on improving the accuracy of the model to make it more practical for deployment-grade performance.

## Figures and Tables

**Table 1:** Classification-based approaches for plant disease identification.

Authors	Network Architecture	Disease Category	Pre-Training Dataset	Fine-Tuning Dataset	No. of Classes	Accuracy(%)
Liu et al. [29]	AlexNet	Apple	ImageNet	Field Collected	4	97.62
Fang et al. [24]	ResNet50	Leaf	-	PlantVillage	27	95.61
Hasan et al. [26]	InceptionV3+SVM	Rice	ImageNet	Field Collected, Online	9	97.5
Dechant et al. [28]	Custom CNNs	Maize	-	Field Collected	2	96.7
Barbedo et al. [30]	GoogLeNet	12 plant species	ImageNet	-	12	87
Ramcharan et al. [31]	InceptionV3	Cassava	ImageNet	Field Collected	5	-
Kawasaki et al. [32]	Modified LeNet	Cucumber	-	Laboratory Collected	3	94.9

**Table 2:** Object detection-based approaches for plant disease detection.

Authors	Network Architecture	Disease Category	Pre-Training Dataset	Fine-Tuning Dataset	No. of Classes	Accuracy(%)
Nie et al. [47]	Faster R-CNN+Attention	Strawberry	ImageNet	Field Collected	4	78.05
Byoungjun et al. [4]	Cascaded Faster R-CNN	Strawberry	PlantCLEF	Field Collected	7	91.62
Ramcharan et al. [48]	SSD	Cassava	MS-COCO	Field Collected	3	-
Ozguven et al. [46]	Modified Faster R-CNN	Sugar beet	-	Field Collected	4	95.48
Fuentes et al. [1]	Faster R-CNN+Filterbank	Tomato	ImageNet	Field Collected	10	96.25
Fuentes et al. [45]	FPN + LSTM	Tomato	ImageNet	Field Collected	10	92.5

**Table 3:** Summary of visual segmentation-based models for monitoring plant diseases.

Authors	Network Architecture	Disease Category	Pre-Training Dataset	Fine-Tuning Dataset	No. of Classes	Accuracy (%)
Stewart et al. [54]	Mask R-CNN	Northern Leaf Blight	MS-COCO	Field Collected	1	96
Lin et al. [58]	Modified U-Net	Cucumber Powdery Mildew	-	Laboratory Collected	1	96.08
Wang et al. [59]	FCN	Maize Leaf Disease	-	Field Collected	6	96.26

**Table 4:** A summary of our dataset.

Category of Disease	Images for Training	Images for Validation	Images for Testing
Angular Leafspot	245	43	147
Anthracnose Fruit Rot	52	12	33
Blossom Blight	117	29	62
Gray Mold	255	77	145
Leaf Spot	382	71	162
Powdery Mildew Fruit	80	12	43
Powdery Mildew Leaf	319	63	151
Total	1450	307	743

**Table 5:** Results on our dataset for ResNet50/101 without using any augmentation technique.

Network	mAP (%)
ResNet50	72.06
ResNet101	71.69

**Table 6:** Details of the various augmentation techniques tested on our dataset.

Augmentation	Specifications	mAP (%)
Baseline	-	71.69
Change Color Temperature	(7000, 12000)	68.92
Dropout	p = (0, 0.2)	71.74
**Edge Detect**	**alpha = (0.0, 1.0)**	**72.37**
**Enhance Color**	-	**72.02**
Filter Edge Enhance	-	68.34
Gamma Contrast	(0.5, 2.0)	71.90
Gaussian Blur	sigma = (0.0, 2.0)	68.70
Histogram Equalization (All Channels)	-	71.02
Multiply	(0.4, 1.4)	70.58
Multiply and Add to Brightness	mul = (0.5, 1.5), add = (−30, 30)	72.26
Multiply Hue and Saturation	(0.3, 1.3), per_channel = True	73.79
Perspective Transform	scale = (0.01, 0.15)	68.90
**Rotate**	**(−45, 45)**	**72.91**
Rotate + Edge Detect	copied from individual application	73.63
Rotate + Enhance Color + Sharpen	copied from individual application	75.88
**Sharpen**	**alpha = (0.0, 1.0), lightness = (0.75, 2.0)**	**70.72**

**Table 7:** Final results on ResNet50 and ResNet101 with updated learning rate schedule.

Network	Augmentation	Improved Training Strategy	mAP (%)
ResNet50	√		79.84
ResNet50	√	√	81.37
ResNet101	√		80.24
ResNet101	√	√	**82.43**

**Table 8:** Per-class Average Precicion for ResNet50/101.

Class	AP for ResNet50 (%)	AP for ResNet101 (%)
Angular Leafspot	79.93	**81.16**
Anthracnose Fruit Rot	**71.46**	63.63
Blossom Blight	**87.90**	82.25
Gray Mold	92.29	**93.90**
Leaf Spot	71.93	**73.33**
Powdery Mildew Fruit	68.02	**70.91**
Powdery Mildew Leaf	85.66	**89.87**

**Table 9:** Performance evaluation for variable disease severity.

Network	Level	Infection Status	mAP IOU 0.50 (%)	mAP IOU 0.50:0.95 (%)
Mask R-CNN	Level 1	Low-Mid	86.10	64.76
Mask R-CNN	Level 2	High	81.02	58.10

**Table 10:** Comparison with other architectures.

Network	Backbone	mAP IOU 0.50 (%)	mAP IOU 0.50:0.95 (%)
Mask R-CNN	ResNet50	81.37	55.21
Mask R-CNN	ResNet101	**82.43**	**59.94**
YOLACT	ResNet50	79.71	55.19
YOLACT	ResNet101	79.39	55.81

**Table 11:** A comparison with relevant literature.

Authors	Network Architecture	Pre-Training Dataset	Fine-Tuning Dataset	No. of Classes	Accuracy (%)	Approach
Ouyang et al. [69]	SVM	-	Field Collected	3	-	Traditional Segmentation
Nie et al. [47]	Faster R-CNN+Attention	ImageNet	Field Collected	4	78.05	Object Detection
Byoungjun et al. [4]	Cascaded Faster R-CNN	PlantCLEF	Field Collected, Online	7	91.62	Object Detection
**This Work**	**Mask R-CNN**	**MS-COCO**	**Field Collected, Online**	**7**	**82.43**	**Fine-grained Instance Segmentation**

## Data Availability

The dataset presented in this study is openly available at www.kaggle.com/usmanafzaal/strawberry-disease-detection-dataset (acessed on 30 September 2021).
